# Safety and Efficacy of Repeat Open-Label AbobotulinumtoxinA Treatment in Pediatric Cerebral Palsy

**DOI:** 10.1177/0883073817729918

**Published:** 2017-09-15

**Authors:** Mauricio R. Delgado, Marcin Bonikowski, Jorge Carranza, Edward Dabrowski, Dennis Matthews, Barry Russman, Ann Tilton, Juan Carlos Velez, Anne-Sophie Grandoulier, Philippe Picaut

**Affiliations:** 1 University of Texas Southwestern Medical Center and Texas Scottish Rite Hospital for Children, Dallas, TX, USA; 2 Mazovian Neuropsychiatry Center, Warsaw, Poland; 3 Hospital San José Celaya, Celaya, Guanajuato, Mexico; 4 Beaumont Health, Oakland University School of Medicine, Grosse Pointe, MI, USA; 5 Children’s Hospital Colorado, Aurora, CO, USA; 6 Shriner’s Hospital for Children, Portland, OR, USA; 7 LSUHSC and Children’s Hospital New Orleans, New Orleans, LA, USA; 8 Centro de Rehabilitacion Club De Leones Cruz del Sur, Punta Arenas, Chile; 9 Ipsen, Les Ulis, France

**Keywords:** abobotulinumtoxinA, Dysport, cerebral palsy, equinus foot

## Abstract

This was a prospective, repeat-treatment, open-label study (NCT01251380) of abobotulinumtoxinA for the management of lower limb spasticity in children who had completed a double-blind study. Children (2-17 years) received injections into the gastrocnemius-soleus complex, and other distal and proximal muscles as required (maximum total dose per injection cycle: 30 U/kg or 1000U). A total of 216 of the 241 double-blind patients entered the extension study and 207 received ≥1 open label injection into the gastrocnemius-soleus; 17-24% of patients also had injections into the hamstrings. The most frequent adverse events were related to common childhood infections and the most frequent treatment-related adverse event was injection site pain (n = 10). There was no evidence of a cumulative effect on adverse events. Sustained significant clinical improvements in muscle tone (Modified Ashworth Scale), spasticity (Tardieu Scale), overall clinical benefit (Physicians Global Assessment), and goal attainment (Goal Attainment Scale) were also observed across treatment cycles.

The long-term utility of botulinum toxin type-A treatment in the management of children with cerebral palsy is well established through years of clinical use. However, the evidence base for botulinum toxin type-A in cerebral palsy has been of variable quality and has primarily been based on studies of single injection cycles.^1^ Repeat injections are necessary to achieve a longer-lasting effect, but the results of repeat injections in pediatric cerebral palsy patients have only been evaluated in a few case series and relatively small clinical trials. Where available, such studies have shown repeat injections in children with dynamic equinus foot deformity to be generally safe and efficacious in improving various aspects of gait,^2–6^ and a recent systematic review found that children with spastic cerebral palsy show functional gains especially after the first 2 injections (ie, 1 repeat injection of botulinum toxin type-A).^7^ However, the review found that there was insufficient evidence to review the effect of multiple injections.^7^

The authors have previously reported the results of a large, international, randomized, placebo-controlled study designed to prospectively assess the efficacy and safety of abobotulinumtoxinA (Dysport^®^; Ipsen Pharma, Wrexham, UK) versus placebo in children with dynamic equinus foot deformity due to cerebral palsy.^8^ This trial was the first large trial specifically designed to assess multidimensional efficacy of botulinum toxin type-A treatment via a broad variety of outcome measures, from muscle tone and spasticity assessments, to overall clinical status and goal achievement. The authors report here the safety and efficacy of repeat abobotulinumtoxinA injection cycles from the open-label extension study. The study continued to assess efficacy utilizing methods that are relevant to patients, their families and health care providers.

## Methods

This was a phase III, prospective, multicenter, repeat treatment, open-label, extension study (NCT01251380), following the double-blind study reported by Delgado et al.^8^ Patients participating in the double-blind study were offered the option of entering the open-label study once a new informed consent form had been signed. The study was conducted between October 2011 and January 2014 in 26 active sites in 6 countries in North and South America, Europe, and Asia. Institutional review boards at the participating sites approved the protocol and the trial was executed in accordance with the Declaration of Helsinki and International Conference on Harmonization Good Clinical Practice Guidelines.

### Patients

The study included ambulatory pediatric patients (2-17 years old) with dynamic equinus foot deformity due to cerebral palsy (Gross Motor Function Classification System levels I-III) who had completed the double-blind study (ie, at least 12 weeks of follow-up after injection) without any major protocol deviations and/or any ongoing adverse events. Full entry criteria for the double-blind study have been previously reported.^8^

### Study Design

This open-label study consisted of a maximum of 4 treatment cycles of abobotulinumtoxinA at doses of 5 to 30 U/kg, occurring at intervals of ≥12 weeks, depending on overall individual duration of response to treatment. The first reinjection (start of open-label cycle 1) was administered at or after the week 12 visit of the double-blind study. If, according to the Investigator’s judgment, the patient did not yet require retreatment at week 12 of the double-blind study, the first reinjection was postponed until he/she did require retreatment (which could be at weeks 16, 22, 28, or later of the double-blind study). Investigators considered the need for reinjection based on treatment response. If the patient did not demonstrate a treatment-response, considered as a decrease from baseline of ≥1 grade in the Modified Ashworth Scale score in the ankle flexor and/or an improvement on the Physician’s Global Assessment (score <0), and there were no safety risks, then they were eligible for reinjection. If the patient did demonstrate a treatment-response, the investigator decided based on the other efficacy and safety assessments whether the patient needed to be injected at that visit or whether the injection was postponed to a later visit.

### Injection Procedures

Injections were guided by electrical stimulation or ultrasound, and centers maintained their usual practice for anesthesia and pain management. In cycle 1, the total dose for unilateral injection into the gastrocnemius-soleus complex (ie, for patients with hemiparesis) was 10 U/kg and the total dose for bilateral injection (ie, patients with diparesis) was 20 U/kg. In patients receiving bilateral injections, the 20 U/kg dose could be split according to the investigator’s judgment between the 2 gastrocnemius-soleus complexes with a minimum of 5 U/kg in 1 leg and a maximum of 15 U/kg in the other leg. As per the double-blind study, all injections into the gastrocnemius-soleus complex were split between the gastrocnemius and soleus muscles at a ratio of 3:2.^8^ In addition, if patients required hamstring injections in cycle 1, the total dose per lower limb was split equally between the hamstrings and ipsilateral gastrocnemius-soleus complex.

From cycle 2 onward, the total dose could be adapted for each individual patient as clinically indicated. Although all patients continued to be injected into the gastrocnemius-soleus complex, individualization of treatment could now also include injections of other lower limb muscles (tibialis posterior, hamstrings, hip adductors [any], or iliopsoas) or upper limb muscles. For bilateral injections, doses could be split unequally between the 2 limbs. The dose per lower limb could have been increased or decreased by no more than 5 U/kg per affected lower limb per cycle (5-20 U/kg/leg), or remain the same. Upper limb injections had to be concomitant to lower limb injections and could not exceed 10 U/kg. The total dose of abobotulinumtoxinA (irrespective of muscles injected) per cycle was no more than 30 U/kg or 1000 U, whichever was the lower value.

Each patient was considered to have completed the study once the total treatment follow-up duration (inclusive of the double-blind study) reached 12 months, and not before their week 4 visit of the last treatment cycle in this open-label study. If physical therapy or ankle-foot orthoses had been initiated prior to double-blind study, the therapy regimen was continued (at the same frequency and intensity) for the duration of treatment. If absolutely required during the open-label phase, new physical therapy, ankle-foot orthoses, and casting were permitted only after the week 12 study visit of the first treatment cycle.

### Assessments

Patients were assessed at weeks 4 and 12 of open-label cycles 1 to 3. After week 16, discretionary visits were possible every 6 weeks. Patients were assessed at week 4 of cycle 4. Treatment-emergent adverse events were recorded at each visit. Clinical laboratory parameters (hematology, clinical chemistry) were recorded at day 1 of cycle 1 and at week 4 of each cycle. In addition, the presence of antibodies against botulinum toxin type-A was assessed at day 1 of cycle 1, week 4 of treatment cycle 2 and at the end of study or early withdrawal.

Efficacy assessments from the double-blind study were continued into the open-label study, and investigators continued to receive assessment training for standardization of outcome measures. Muscle tone and spasticity were assessed using the Modified Ashworth Scale and the Tardieu Scale, respectively (see the Supplementary Appendix). Since hamstring injections were now permitted (in addition to gastrocnemius-soleus complex injections), both these assessments were performed for the ankle and knee flexors. In addition, patient functionality was assessed using the Physician’s Global Assessment and goal attainment scaling,^9^ as previously described.^8^

### Analyses

The primary objective of this study was the evaluation of abobotulinumtoxinA safety after repeat injections over 1 year. Only descriptive analyses were performed for safety and efficacy parameters; no formal statistical significance testing was planned.

Safety evaluations were performed on the safety population (all enrolled patients) and efficacy analyses (mean changes from the baseline of the double-blind study for all doses of abobotulinumtoxinA combined) were performed in the intent to treat population.

## Results

### Patient Disposition and Baseline Characteristics

Of the 241 patients randomized into the double-blind study, 216 (90%) entered the open-label extension study at time points determined by their eligibility for retreatment during the double-blind phase. Overall, the majority (74%) of abobotulinumtoxinA treated patients did not meet retreatment criteria until at least the week 16 visit; of these, 17.7% did not meet retreatment criteria until week 28 or later.

**Figure 1.**
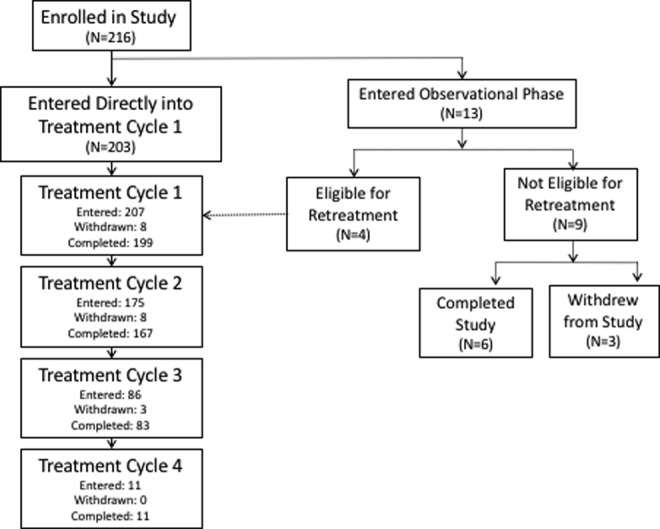
Patient disposition.

Of the 216 patients who entered in the open-label study, 203 entered directly into treatment cycle 1 while 13 patients (all on active treatment) entered an observational phase as they still did not fulfill retreatment criteria. During this observational phase, 4 patients eventually met retreatment criteria while 9 patients were never retreated. Ultimately, a total of 207 patients received ≥1 abobotulinumtoxinA injection cycle (175 had 2 injections, 86 had 3 injections and only 11 patients required a fourth injection) (Figure 1).

Baseline demographics and clinical characteristics are summarized in Table 1. There were no notable differences in patient demographics according to the treatment received in the double-blind study. Approximately 56% of patients were reported as having casting/orthoses during the study (of these <7% were casted), and 75% had physical therapy.

**Table 1.** Baseline Demographics and Clinical Characteristics (Safety Population).

	All patients (N = 216)
Sex, n (%)	
Male	130 (60.2)
Female	86 (39.8)
Age (years), mean ± SD	5.9 ± 3.3
2-9, n (%)	183 (84.7)
10-17, n (%)	33 (15.3)
Pattern of paresis, n (%)	
Hemiparesis	111 (51.4)
Diparesis	93 (43.1)
Tetraparesis	9 (4.2)
Paraparesis	3 (1.4)
GMFCS level, n (%)	
I	128 (59.3)
II	67 (31.0)
III	21 (9.7)
Presence of athetoid or dystonic movements, n (%)	20 (9.3)
History of epilepsy, n (%)	21 (9.7)
Taking antiepileptic medications, n (%)	12 (5.6)
Derived MAS score, mean ± SD	3.1 ± 0.3

MAS is displayed on a derived scale where a score of 0 = 0, 1 = 1, +1 = 2, 2 = 3, 3 = 4, and 4 = 5. GMFCS, Gross Motor Function Classification System; MAS, Modified Ashworth Scale.

### Treatment exposure

All patients had abobotulinumtoxinA injections into the gastrocnemius-soleus complex. Table 2 provides the median and range of doses used per muscle per leg. Overall, 9-11% of patients had injections into the upper limb muscles during cycles 2 and 3.

**Table 2.** Muscle Groups Injected (Safety Population).

	Open-label			
	Cycle 1	Cycle 2	Cycle 3	Cycle 4
Number of patients*	n = 117 unilateral	n = 98 unilateral	n = 42 unilateral	n = 3 unilateral
	n = 84 bilateral	n = 70 bilateral	n = 38 bilateral	n = 5 bilateral
Number of legs injected	285	238	118	13
Muscle				
Gastrocnemius-soleus complex	284 (99.6%), 10.0 [3.5-16.0]	238 (100%), 10.0 [2.5-15.0]	118 (100%), 10.0 [3.9-17.0]	13 (100%), 10.0 [7.5-15.0]
Hamstrings	51 (17.9%), 5.0 [2.0-10.0]	64 (26.9%), 7.5 [2.5-10.0]	26 (22.0%), 5.3 [1.5-10.0]	2 (15.4%)**, 7.5
Tibialis posterior	—	25 (10.5%), 4.0 [1.2-7.5]	8 (10.0%), 4.1 [2.3-8.3]	—
Hip adductors	—	6 (2.5%), 2.5 [2.3-6.5]	2 (1.7%)**, 3.8	—
Iliopsoas	—	1 (0.4%), 2.0	2 (1.7%)**, 1.9	—
Other lower limb muscle	—	3 (1.3%), 4.0 [2.0-18.0]	—	—

Values are number (%) legs injected, median [range] dose (U/kg/leg). *Average dosing data are presented per leg and not per patient because doses for bilateral injections could be split unequally between the 2 legs. **1 patient injected bilaterally into relevant muscle (ie, 2 legs).

### Safety

During the study, 150 (73.5%) patients reported ≥1 treatment-emergent adverse event, of which the majority were of mild intensity and not related to the study treatment. The most frequently reported treatment-emergent adverse events were the common childhood infections nasopharyngitis and upper respiratory tract infection, and associated pyrexia. The proportion of patients reporting treatment-emergent adverse events tended to decrease with each treatment cycle and the incidence of treatment-related treatment-emergent adverse events was low, with no apparent differences in incidence per dose or the number of limbs injected (Table 3). The only treatment-related treatment-emergent adverse event reported by >2 patients was injection site pain, for which 11 instances were reported by 10 patients. There were no differences in the number of treatment-related treatment-emergent adverse events according to the dose administered, and no trend toward a greater frequency of events with increasing number of cycles.

**Table 3.** Adverse Events (AEs) Throughout Open-Label Cycles (Safety Population).

Adverse event, n (%)	Cycle 1 (n = 201)	Cycle 2 (n = 168)	Cycle 3 (n = 80)	Cycle 4 (n = 8)
Any treatment-emergent AE	82 (40.8)	46 (27.4)	14 (17.5)	1 (12.5)
Any treatment-related AE	16 (8.0)	4 (2.4)	2 (2.5)	1 (12.5)
Treatment related AEs				
Injection site pain	7 (3.5)	3 (1.8)	1 (1.3)	—
Injection site papule	2 (1.0)	1 (0.6)	1 (1.3)	—
Influenza like illness	2 (1.0)	—	—	—
Pyrexia	1 (0.5)	—	—	—
Injection site erythema	1 (0.5)	—	—	—
Injection site hemorrhage	1 (0.5)	—	—	—
Fecal incontinence	1 (0.5)	1 (0.6)	—	—
Diarrhea	1 (0.5)	—	—	—
Pain in extremity	1 (0.5)	1 (0.6)	—	—
Urinary incontinence	—	1 (0.6)	—	—
Fall	—	1 (0.6)	—	—
Laceration	—	1 (0.6)	—	—
Phlebitis	—	—	—	1 (12.5)

Seven (3.3%) patients reported a serious treatment-emergent adverse event, all of which were considered unrelated to study treatment. Two patients reported seizures during the open-label study; 1 patient (aged 7), who had prior history of epilepsy, experienced seizures during cycles 1 (total abobotulinumtoxinA dose 10 U/kg) and 2 (total abobotulinumtoxinA dose 20 U/kg) of the open-label study. The second patient (aged 3), who did not have any prior history of seizures, experienced 2 partial seizures during cycles 1 (total abobotulinumtoxinA dose 20 U/kg) and 2 (total abobotulinumtoxinA dose 30 U/kg) of the open-label study. In both patients these events were assessed by the Investigator as unrelated to study treatment. Only 1 patient withdrew from the study due to a treatment-emergent adverse event (pineal gland cyst), which was considered unrelated to study treatment. Evidence of seroconversion to neutralizing antibodies was found in 4 of 193 abobotulinumtoxinA-treated patients analyzed (2.1%) without any associated efficacy or safety concerns. All 4 patients had been previously treated with botulinum toxin type-A before entering the double-blind study and received either 3 or 4 injections during the double-blind and open-label phases.

### Efficacy

#### Hypertonia and spasticity

The efficacy of abobotulinumtoxinA in the treatment of lower limb spasticity seen in the double-blind phase was maintained across the first 3 open-label cycles. Too few patients (n = 11) entered cycle 4 to draw conclusions.

**Figure 2.**
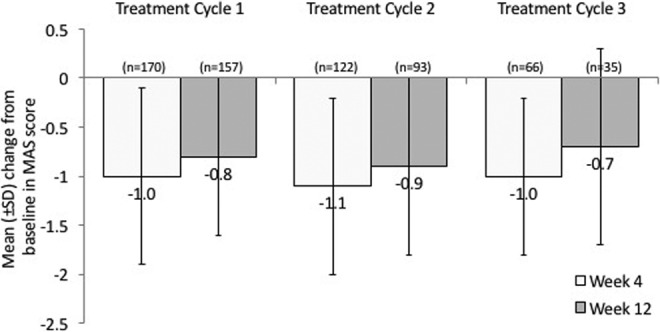
Change from baseline to week 4 and week 12 in Modified Ashworth Scale scores at the ankle plantar flexors.

The magnitude of improvement on Modified Ashworth Scale (approximately 1 grade) was similar across cycles 1-3, and was comparable to that seen in the abobotulinumtoxinA treatment groups in the double-blind study (Figure 2). Clinical improvements in Modified Ashworth Scale scores were also seen at week 12 in all 3 treatment cycles. In those patients who received proximal injections, treatment with abobotulinumtoxinA also improved muscle tone in the knee flexors (Table 4). For patients treated in the upper limb, there was a consistent improvement in the Modified Ashworth Scale for both the elbow flexors and wrist flexors at weeks 4 and 12 in both treatment cycles (see the Supplementary Appendix for full data).

**Table 4.** Efficacy Assessments in the Most Affected Leg.

Outcome measure	Cycle 1	Cycle 2	Cycle 3
Modified Ashworth Scale scores in knee flexors			
N	25	33	13
Mean ± SD change at week 4	–0.6 ± 0.7	–0.5 ± 0.9	–0.2 ± 0.8
N	24	21	5
Mean ± SD change at week 12	–0.8 ± 0.8	–0.3 ± 0.9	–0.6 ± 0.5
Tardieu scale angle of arrest (X_V1_) in ankle plantar flexors			
N	169	121	66
Mean ± SD change at week 4	2.6 ± 9.0	1.4 ± 9.2	0.5 ± 10.1
N	156	92	35
Mean ± SD change at week 12	1.4 ± 9.2	0.4 ± 10.9	–1.1 ± 10.4
Tardieu scale angle of catch (X_V3_) in ankle plantar flexors			
N	168	119	65
Mean ± SD change at week 4	12.2 ± 13.7	13.3 ± 12.6	12.7 ± 13.4
N	155	92	35
Mean ± SD change at week 12	9.9 ± 13.7	11.4 ± 13.9	9.9 ± 14.5
Tardieu scale spasticity angle (X) in ankle plantar flexors			
N	168	119	65
Mean ± SD change at week 4	–9.7 ± 12.6	–12.0 ± 12.0	–12.2 ± 11.6
N	155	92	35
Mean ± SD change at week 12	–8.5 ± 11.9	–10.9 ± 12.8	–11.0 ± 13.4
Tardieu scale spasticity grade (Y) in ankle plantar flexors			
N	169	120	66
Mean ± SD change at week 4	–0.4 ± 0.7	–0.4 ± 0.7	–0.4 ± 0.6
N	155	92	35
Mean ± SD change at week 12	–0.3 ± 0.6	–0.3 ± 0.6	–0.3 ± 0.6
Goal attainment scaling *t*-score*			
N Mean ± SD score at week 4	194 50.6 ± 11.0	158 51.2 ± 11.0	78 48.3 ± 10.7
N	178	116	36
Mean ± SD score at week 12	50.7 ± 10.1	51.7 ± 10.5	45.8 ± 8.9
Physician’s Global Assessment scores			
N	195	159	78
Mean ± SD score at week 4	1.5 ± 0.9	1.5 ± 1.0	1.4 ± 0.9
N	180	116	37
Mean ± SD score at week 12	1.0 ± 1.2	0.9 ± 1.3	0.7 ± 1.4
Modified Ashworth Scale responders (% patients achieving ≥1 grade improvement)			
N	170	122	66
N (%) at week 4	121 (71.2)	90 (73.8)	43 (65.2)
N	157	93	35
N (%) at week 12	96 (61.1)	60 (64.5)	15 (42.9)

Dose ranges were >7.5 to ≤17.5u/kg. Patients with dosages outside of these ranges were excluded from the analysis. *A goal attainment scaling *t*-score greater than 50.0 represents a better than expected outcome.

Similar reductions in spasticity of the gastrocnemius-soleus complex were also observed to those in the double-blind study^8^ for the Tardieu scale angle of arrest (X_V1_), angle of catch (X_V3_), spasticity angle (X) and spasticity grade (Y). Improvements observed at week 4 for X_V3_, X, and Y were maintained at week 12 (Table 4). Similar spasticity results were seen in the knee flexors for patients that were injected in the proximal muscles (Supplementary Appendix).

#### Goal attainment scaling and physician global assessment of treatment response

The 3 most frequently chosen goals in cycle 1 and 2 were improved walking pattern, improved balance and decreased frequency of falling (Supplementary Appendix). In cycle 1 and 2, the proportion of patients choosing “improved walking pattern” and “improved balance” were like that in the double-blind study. However, in cycle 3, the proportion of patients choosing the goal of “improved balance” was higher than in the previous treatment cycles (47.5% in cycle 3 versus 29.2% in cycle 1 and 32.9% in cycle 2). Goal attainment scaling *t*-scores at week 4 and 12 in cycle 1 and cycle 2 were >50.0 indicating a better than expected outcome. In treatment cycle 3 at week 4 and 12 there was a lower than expected outcome (goal attainment scaling *t*-score of 48.3 and 45.8 respectively).

Mean Physician’s Global Assessment score at week 4 and 12 was like that seen in the abobotulinumtoxinA treatment groups in the double-blind study. The magnitude of the response was similar across all cycles, with a trend toward a better response at the higher 15 U/kg/leg dose.

## Discussion

The authors report here the long-term safety and efficacy results following repeated injections of abobotulinumtoxinA in children with dynamic equinus foot deformity due to spasticity in cerebral palsy who were enrolled in the largest double-blind, placebo-controlled study conducted to date.^8^ AbobotulinumtoxinA was well tolerated after repeated injections at total doses from 5 U/kg injected unilaterally to 30 U/kg injected bilaterally, with maximal total dose of 1000 U. The beneficial effects in reducing tone and spasticity observed in the active treatment arms of the double-blind study were maintained throughout the 1-year open-label study.

There was no evidence of a cumulative effect on adverse events with repeated administration of abobotulinumtoxinA. As per the double-blind study,^8^ the most frequent treatment-emergent adverse events were common childhood infections (upper respiratory tract infections). The pattern of treatment-emergent adverse events and causally related treatment-emergent adverse events observed was similar between treatment cycles and were also consistent with previous double-blind clinical study. There were no deaths reported or treatment related serious adverse events. Specifically, there was no increased incidence of muscle weakness or atrophy reported in this study. Only 1 patient out of 21 who had a history of epilepsy had seizures during the study. One patient without prior history of seizures had new onset of seizures; neither case was considered related to study treatment.

To date, there has been a distinct lack of studies evaluating the long-term efficacy of repeat treatment in this group of patients. The present results indicate that the efficacy seen in the double-blind study^8^ was maintained through repeated injections. In terms of hypertonia and spasticity (Modified Ashworth Scale and Tardieu scale), the magnitude of improvements and percentage of responders in cycles 1-3 were like that reported in the double-blind study.^8^ Patients who had knee flexors injected showed a similar improvement in hypertonia and spasticity. Goal attainment and the Physician’s Global Assessment of treatment response were also improved in a consistent manner to the double-blind study. It is, however, interesting to note that there was a shift in goal selection in cycle 3, where a higher proportion of patients had “improved balance” as a goal of treatment. As seen in the double-blind study this goal is harder to achieve,^8^ and the shift in goal setting could explain why cycle 3 goal attainment scaling *t*-scores indicated less than expected goal achievement. It may be that some patients may have achieved expected improvements in walking pattern in previous cycles and set the harder to achieve balance goal as the next step in their therapy journey. Conversely, the lower goal attainment scaling *t*-scores could also reflect the fact that cycle 3 is skewed toward patients who required further treatment, as those who did not require retreatment did not enter this cycle.

One of the key strengths of this study (and the preceding double-blind study) was the considerable emphasis placed on good injection technique using electrical stimulation or ultrasound as guidance, and on standardization of assessments. All investigators had undergone a rigorous training program to perform Modified Ashworth Scale and Tardieu Scale assessments in the same way. Additional strengths of this study lie in the size (90% of patients in the double-blind study continued into the open-label study) and duration (1-year) of the study, the multidimensional approach to efficacy assessment and the multicenter (multicultural) approach. Injectors were given the freedom to tailor injections (within dosing limits of the protocol) to the needs and goals of the patient by injecting proximal leg muscles and upper limb muscles as clinically indicated.

As with the double-blind study, limitations of this open-label study include the skew to milder cerebral palsy and the absence of patients with severe cerebral palsy (Gross Motor Function Classification System levels IV–V) who were excluded because this study was designed for ambulatory children. Only 11 patients entered cycle 4, restricting the usefulness of any analyses for this cycle. This is primarily because most patients did not require a fourth retreatment within the open-label follow-up period as they were still deriving benefit from the previous treatment cycles. The fact that 1 in 5 patients did not require reinjection for at least 28 weeks after the double-blind phase supports the idea that the therapeutic benefits of chemodenervation with abobotulinumtoxinA lasts longer than the accepted “12-week” pharmacologic action of botulinum toxin type-A at the neuromuscular junction. While the number of patients receiving injections in the upper limb (as well as lower limb) was limited (n = 21), muscle tone (Modified Ashworth Scale scores) in the upper limb also tended to improve. A double-blind, randomized placebo-controlled study (NCT02106351) has started to prospectively evaluate the efficacy of abobotulinumtoxinA in this indication.

## Conclusion

In this open-label study, the safety profile of abobotulinumtoxinA injected at doses up to 30 U/kg (or a maximum of 1000 U) was consistent with the known safety profile of abobotulinumtoxinA in children with lower limb spasticity due to cerebral palsy and no additional safety concerns were identified. Treatment with abobotulinumtoxinA administered in accordance with the pattern of impairment (distal and proximal muscles) and treatment objectives led to sustained clinical improvements in hypertonia, overall clinical benefit, and goal attainment over 1-year of repeat injections. These data confirm the favorable benefit-risk ratio for abobotulinumtoxinA in the treatment of children with lower limb spasticity.
